# The critical issue of grade 2 vs grade 3 in IDH-mutant gliomas

**DOI:** 10.1093/neuonc/noag143

**Published:** 2026-06-23

**Authors:** Ho-Keung Ng

**Affiliations:** Department of Anatomical & Cellular Pathology, Chinese University of Hong Kong, Hong Kong, China (H.-K.N)

In the pre-IDH-inhibitor era, grading an IDH-mutant glioma pathologically as grade 2 or grade 3 could largely be a prognostic and risk stratification issue, as international guidelines at the time endorsed both approaches of watchful waiting and RT-chemotherapy for a grade 2 IDH-mutant glioma after resection.^1^ Most patients with grade 2 IDH-mutant gliomas have good PFS in the first few years after resection, and it is mostly by a grade 3 rather than grade 4 histology that the tumor first undergoes malignant transformation, after which survival becomes compromised.^2^ A few studies, controversially, did not show a significant survival difference between histological grade 3 and grade 2 IDH-mutant gliomas, although the lack of standardized criteria for grading could be a problem in some studies.^3-5^ After the publication of the INDIGO trial,^6^ FDA approved in 2024 the use of vorasidenib in patients with grade 2 IDH-mutant astrocytoma and oligodendroglioma after resection. In practice, a diagnosis of grade 2 gives the patient and neuro-oncologist the option of vorasidenib treatment, and grade 3 generally precludes its use. It is unknown for the present whether IDH-inhibitors will clearly benefit patients with grade 3 tumors, and studies are on-going. So the issue of distinguishing between grade 2 and grade 3 pathologically now has become critical for IDH-mutant gliomas.

Since the WHO (2021) classification, CDKN2A HD (homozygous deletion) has been included in the molecular assessment of IDH-mutant gliomas. WHO (2021) established CDKN2A HD as a diagnostic criterion for grade 4 IDH-mutant astrocytoma when necrosis or microvascular proliferation was absent. However, it is not always appreciated that CDKN2A HD is a diagnostic criterion for grade 4 IDH-mutant astrocytoma only, and that there is actually no molecular criteria for distinguishing grade 3 from grade 2 IDH-mutant gliomas. The key publications on which the recommendation was based showed the prognostic significance of CDKN2A HD on mostly grade 3 IDH-mutant astrocytomas and grade 3 oligodendrogliomas.^7^^,^^8^ For any individual tumor, if the histology is borderline and equivocal, there are currently no molecular criteria to help make the clinically important distinction of grade 3 vs grade 2 outside histological assessment.

In the current WHO classification, diagnosis of grade 3 IDH-mutant astrocytoma and oligodendroglioma is based on histological features only. Therefore, in practice, the critical differentiation of grade 2 vs grade 3 in IDH-mutant gliomas, where a potentially beneficial target drug can be prescribed or likely excluded, currently depends largely on “eyeball” assessment by the pathologists.^9^ In this issue of Neuro-oncology, Brat et al^5^ writing in the form of a cIMPACT-NOW update, provide a comprehensive review of the risk stratification and pathological grading of IDH-mutant gliomas. One of the issues the authors focus spot on is the grading of WHO grade 3 vs grade 2. Based on their extensive and critical review of corroborative evidence in the literature, the authors now recommend that a diagnosis of IDH-mutant astrocytoma, grade 3, should be made by the presence of mitotic rates of ≥3 per 2.4 mm^2^ or the presence of any from a list of molecular alterations including CDK4, CDK6, CCND2 amplification, RB1 (homozygous deletion or mutation), MYCN, EGFR amplification, PIK3CA or PIK3R1 mutation, and CDKN2A mutation or methylation signature of A_IDH_HG or G-CIMP-low. These molecular alterations correspond to an intermediate risk. As highlighted by Brat et al^5^ many of these molecular changes are part of the RB pathway. IDH-mutant astrocytomas showing PDGFRA amplification or CDKN2A HD but not otherwise showing necrosis, or microvascular proliferation, should be designated grade 4, and PDGFRA change is a new addition to the WHO criteria for grade 4. The recommendations from this paper are likely to be included in the upcoming WHO Classification of CNS Tumors expected to be in print at the end of 2026 or early 2027. These recommendations will serve the diagnostic laboratories well, until further and better evidence hopefully arrives in the future.

For IDH-mutant, 1p19q-codeleted oligodendrogliomas, after a literature review, the authors recommend no changes to the official WHO grading criteria for grade 3: necrosis or microvascular proliferation, or elevated mitotic rates (recommended cutoff: ≥ 6 mitoses/2.4 mm^2^) with no definite molecular grading criteria. There have not been many ground-breaking papers for molecular prognostication in oligodendrogliomas in the last few years. For WHO (2021), CDKN2A HD has not been fully included as a diagnostic criterion for grade 3 oligodendrogliomas. The incidence of CDKN2A HD in grade 3 oligodendrogliomas is low and not frequent enough for this test to be used as diagnostic criteria.^5^ For the cut-off mitotic rate, according to their review, the authors continue to recommend ≥ 6 mitoses per 2.4 mm^2^ as noted by the WHO (2021). Examining for CDKN2A homozygous deletion will be useful, but the alteration is probably not present frequently enough for routine use. There is no grade 4 for oligodendroglioma. The authors note from the literature though that patients with grade 3 oligodendrogliomas diagnosed by elevated mitotic counts only in the absence of MRI-contrast enhancement, necrosis, microvascular proliferation or CDKN2A HD may have better survival.^10^ Whether these features will form the basis of further studies to refine the grading scheme for oligodendrogliomas remains to be seen.

The main concern for implementation of these recommendations lies in the availability of the tests recommended. To pursue the complete panel of molecular tests as recommended by cIMPACT-NOW-12 will require NGS (next generation sequencing) as well as methylation array. While commercial sources for NGS and methylation profiling are available in many parts of the LMICs (low- and middle-income countries), the lack of national funding for these tests in many LMICs may hamper the implementation of these criteria worldwide, and the authors of this paper are fully aware of this issue. There is also an issue of timeliness for reporting an integrated diagnosis. Although claims have often been made for the time efficiency of methylation array and some NGS platforms, real-world workflow in laboratories must also take into account the time needed for additional cutting of slides, transfer (sometimes across national boundaries), analysis, and communications, and these make up in total a multi-step process. It should be noted though, that the majority of the molecular alterations recommended in this cIMPACT-NOW update are copy number changes, in the form of amplification or homozygous deletion, and mutations of PIK3CA and PIK3R1 are usually infrequent as detailed by the paper. Therefore, a case can be made for performing a near-panel of FISH tests in real-world situations, where NGS and methylation array are not available. FISH may be imperfect in this context as it does not examine whole chromosome change. However, FISH is available to most pathology laboratories even in LMICs, due to the need for HER-2 testing for breast cancer, a much commoner cancer than glioma. FISH takes only 2 to 3 days and requires only 1 section; it also does not require skills for analyzing genomic data which may not be available in the regular histology laboratories. A panel of FISH tests for multiple genes included in Brat et al.’s recommendation will also improve diagnostic accuracy. Many of the studies concerning these molecular changes from which the authors have derived their recommendations were also performed with FISH.

A minor issue is the infrequent availability of G-CIMP testing worldwide. In practical terms, the A_IDH_HG and A_IDH_LG distinction, which is also methylation-based and widely used globally, is likely to take precedence over the G-CIMP status.
